# NMGMDA: a computational model for predicting potential microbe–drug associations based on minimize matrix nuclear norm and graph attention network

**DOI:** 10.1038/s41598-023-50793-y

**Published:** 2024-01-05

**Authors:** Mingmin Liang, Xianzhi Liu, Qijia Chen, Bin Zeng, Lei Wang

**Affiliations:** 1 School of Information Engineering, Hunan Vocational College of Electronic and Technology, Changsha, 410000 China; 2 Big Data Innovation and Entrepreneurship Education Center of Hunan Province, Changsha University, Changsha, 410022 China

**Keywords:** Biological techniques, Bioinformatics

## Abstract

The prediction of potential microbe–drug associations is of great value for drug research and development, especially, methods, based on deep learning, have been achieved significant improvement in bio-medicine. In this manuscript, we proposed a novel computational model named NMGMDA based on the nuclear norm minimization and graph attention network to infer latent microbe–drug associations. Firstly, we created a heterogeneous microbe–drug network in NMGMDA by fusing the drug and microbe similarities with the established drug–microbe associations. After this, by using GAT and NNM to calculate the predict scores. Lastly, we created a fivefold cross validation framework to assess the new model NMGMDA's progressiveness. According to the simulation results, NMGMDA outperforms some of the most advanced methods, with a reliable AUC of 0.9946 on both MDAD and aBioflm databases. Furthermore, case studies on Ciprofloxacin, Moxifoxacin, HIV-1 and Mycobacterium tuberculosis were carried out in order to assess the effectiveness of NMGMDA even more. The experimental results demonstrated that, following the removal of known correlations from the database, 16 and 14 medications as well as 19 and 17 microbes in the top 20 predictions were validated by pertinent literature. This demonstrates the potential of our new model, NMGMDA, to reach acceptable prediction performance.

## Introduction

Microorganisms are a class of widely dispersed germs that include bacteria, viruses, fungus, and other species that are both helpful and hazardous to humans^1,2^. Numerous human organs contain and are covered in human microbes^3^. In addition to promoting food absorption and maintaining intestinal health by managing the balance of the gut microbiota, they can control the host's mucosal and systemic immune systems^4,5^. In the intestinal environment, these bacteria depend on one another and benefit one another. When the microbiota is out of balance, several diseases, include obesity^6^, inflammatory bowel disease^7^, and cancer^8^ can result. Additionally, numerous studies have demonstrated that while utilizing pharmaceuticals to cure diseases, there is a definite influence between bacteria and drugs^1,9,10^. Therefore, understanding the relationship between microbes and medications becomes essential for the treatment of disease.

Humans have discovered certain relationships between drugs and microbes through investigations into biology, but because biological experiments demand a substantial amount of human, material, and time resources, their further advancement may be constrained. To address the limitations of biological studies, an increasing number of computational methods have been presented during the past few years due to the rapid development of relevant research tools. These methods aim to anticipate the relationship between drugs and microbes^11^. In parallel, databases of microbe–drug associations that have undergone experimental validation, such as MDAD^12^ and aBioflm^13^, have also been established. For instance, Zhu et al.^14^ presented HMDAKATZ, which uses the KATZ measure to identify microbe–drug associations. By integrating a network embedding approach with microbe–drug association prediction, Long et al.^15^ introduced the HNERMDA method. To predict probable microbe–drug associations, Ma et al.^16^ introduced the generalized Matrix decomposition method WHGMF based on weighted hypergraph learning. To infer new microbe–drug relationships, Yang et al.^17^ suggested the multi-core fusion model MKGNN based on Graph Convolutional Network(GCN). A deep neural network-based prediction model for microbe–drug associations called NNAN was created by Zhu et al.^18^ A contrastive learning model called SCSMDA was created by Tian et al.^19^ to forecast the connection between microbes and drugs. In order to anticipate probable microbe–drug correlations, Tan et al.^20^ developed a computation technique termed GSAMDA based on the graph attention network and the sparse autoencoder. Yang et al.^21^ suggest a model, called MKGCN, for inferring microbe–drug associations based on Multiple Kernel Fusion on Graph Convolutional Network. Ma et al.^22^ designed a microbe–drug prediction model based on graph attention network (GAT) and convolutional neural networks (CNN).

As mentioned above, it is easy to know that these neural network-based methods are frequently used in hiding random association prediction works, and among them, CNN-based approaches adopt the method of parameter sharing to effectively prevent overfitting, however, the pooling layer will lose a significant amount of important data during processing. As for the GCN-based approaches, although the non-matrix organized data will be more applicable, however, the scalability and flexibility are still quite limited. As for the GAT-based methods, although the clustering performance of graph neural networks can be significantly improved, but the clustering of higher-order neighborhoods is still a challenging task. Hence, it is clear that better prediction results can be obtained by combining these above prediction methods organically.

**Figure 1:**
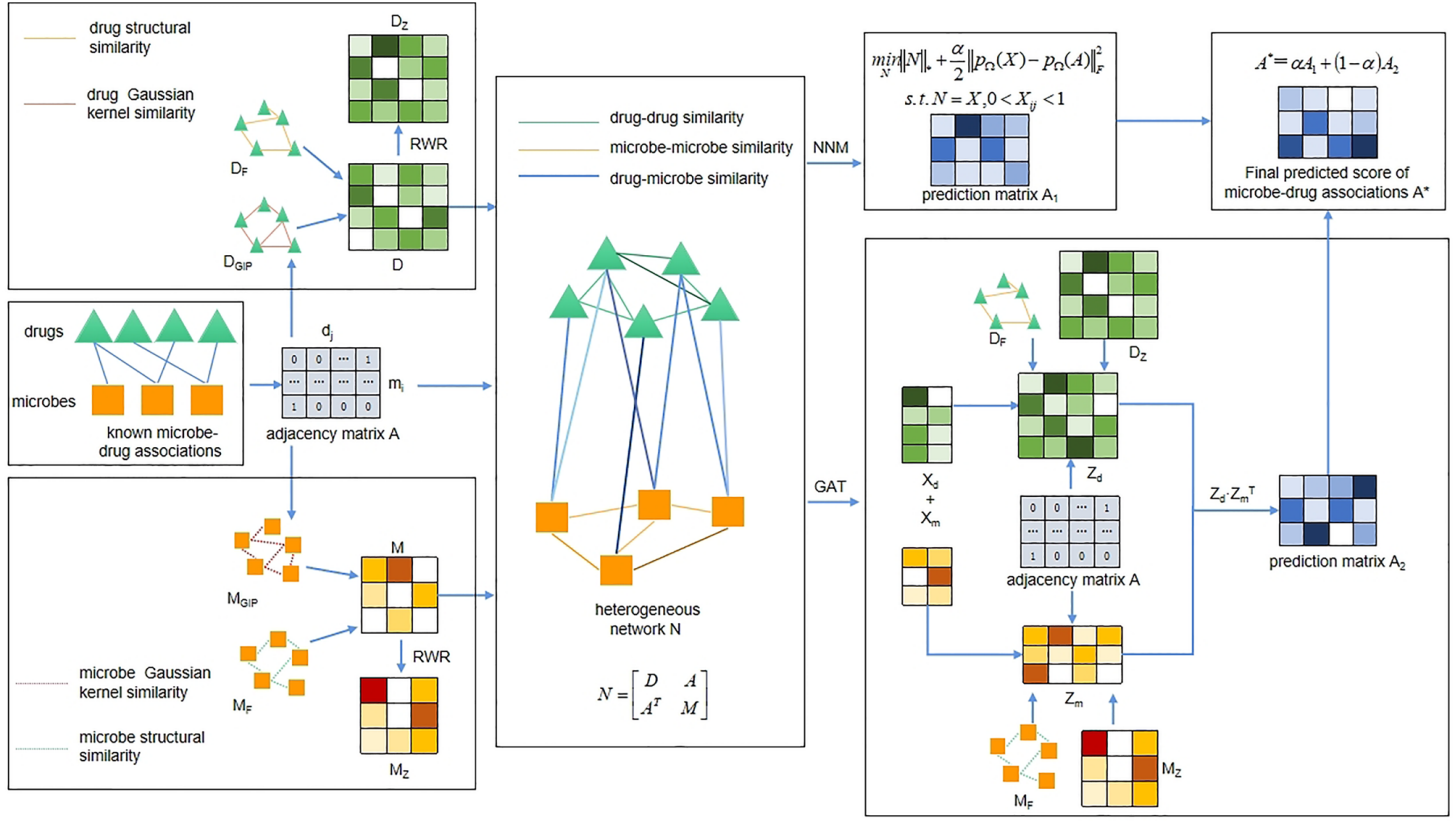
The overall architecture of NMGMDA.

In this study, we introduced a novel calculating approach called NMGMDA to predict latent associations between microbes and drugs, which is based on the nuclear norm minimization^23^ and the graph attention network^24^. Figure 1 depicts the NMGMDA structure. These are our primary contributions, in brief:

- A novel heterogeneous network made up of microbes and drugs has been created by combining the microbe similarity network, drug similarity network, and existing microbe–drug relationships.
- To get projected scores for potential microbe–drug associations, we used both the nuclear norm minimization (NNM) approach and the GAT-based auto-encoder. And then weighted averaged these two predicted scores to get the final results.
- Experimental results and case studies demonstrated the significant prediction performance of NMGMDA on both the MDAD and the aBioflm Databases.

## Materials and methods

### Data sources

In this study, we assessed NMGMDA on the following two databases in order to show its efficacy.

*MDAD database* is a database of microbe–drug associations that was assembled and arranged by Sun et al.^12^ in 2018 from a variety of drug-related databases, including TTD and DrugBank, as well as a substantial body of literature. After superfluous data is eliminated, 1373 drugs and 173 microbes were found to have 2470 microbe–drug associations.

*ABiofilm database* was created by Rajput et al.^13^, which includes 5027 antifungal drugs that target 140 microbes that were identified between 1988 and 2017. Following the removal of redundant data, 140 microbes and 1720 drugs were included in 2884 microbe–drug associations.

Table 1 provides specific statistics of microbes-drugs associations in the MDAD and aBioflm.

**Table 1:** The specific statistics of microbes-drugs associations in the MDAD and aBioflm.

Database	Microbes	Drugs	Associations
MDAD	173	1373	2470
aBioflm	140	1720	2884

## Methods

### Microbe–drug adjacency matrix

We initially create an adjacency matrix $A\in {R}^{{n}_{d}\times {n}_{m}}$, where ${n}_{d}$ and ${n}_{m}$ represent the number of drugs and microbes, respectively, based on these microbe–drug associations. ${A}_{ij}$ equals 1 if there is a known relationship between the drug ${d}_{i}$ and microbe ${m}_{j}$, else it equals 0.

1 $${A}_{ij}=\left\{\begin{array}{l}1, \,\,if \,\,{d}_{i} \,\,associats \,\,with\,\, {m}_{j}\\ 0, \,\,otherwise\end{array}\right.$$

### Drug/microbe Gaussian kernel similarity

The following formula will be used to determine the Gaussian kernel similarity ${D}_{GIP}\left({d}_{i},{d}_{j}\right)\in {R}^{{n}_{d}\times {n}_{d}}$ between ${d}_{i}$ and ${d}_{j}$, assuming that ${d}_{i}$ and ${d}_{j}$ are two drugs.

2 $${D}_{GIP}=exp\left(-{{\gamma }_{d}\Vert A\left({d}_{i}\right)-A\left({d}_{j}\right)\Vert }^{2}\right)$$

where $\Vert A\left({d}_{i}\right)-A\left({d}_{j}\right)\Vert$ is the Euclidean distance between two drugs. Since ${\gamma }_{d}$ is a regular parameter, it is easier to group together similar feature points the greater ${\gamma }_{d}$. And the definition of ${\upgamma }_{{\text{d}}}$ is as follows:

3 $${\gamma }_{d}=1/\left(\frac{1}{{n}_{d}}\sum_{i=1}^{{n}_{d}}{\Vert A\left({d}_{i}\right)\Vert }^{2}\right)$$

Similarly, we would calculate the Gaussian kernel similarity ${M}_{GIP}\left({m}_{i},{m}_{j}\right)\in {R}^{{n}_{m}\times {n}_{m}}$ between two microbes:

4 $${M}_{GIP}=exp\left(-{{\gamma }_{m}\Vert A\left({m}_{i}\right)-A\left({m}_{j}\right)\Vert }^{2}\right)$$

5 $${\gamma }_{m}=1/\left(\frac{1}{{n}_{m}}\sum_{i=1}^{{n}_{m}}{\Vert A\left({m}_{i}\right)\Vert }^{2}\right)$$

### Microbe/Drug functional similarity

In the STRING^25^ database, we can find many gene functional networks connected to microbes. A matrix ${M}_{F}\in {R}^{{n}_{m}\times {n}_{m}}$ can be produced by the Kamneva^26^ tool, which determines microbe functional similarity based on microbial gene families.

The SIMCOMP2 tool^27^ uses the chemical and molecular formula structures of drugs to determine how similar their structures are. To create a drug functional similarity matrix ${D}_{F}\in {R}^{{n}_{d}\times {n}_{d}}$, we adopt the similarity scores.

### Drug/microbe integrated similarities

It is important to note that not every drug can determine functional similarity. As a result, using the drug structural similarity and the drug Gaussian kernel similarity, we were able to construct a new matrix $D\in {R}^{{n}_{d}\times {n}_{d}}$ of integrated drug similarities.

6 $$D=\left\{\begin{array}{c}\left({D}_{GIP}+{D}_{F}\right)/2, if {D}_{F}\ne 0\\ {D}_{GIP}, if {D}_{F}=0\end{array}\right.$$

where ${D}_{GIP}$ is the drug Gaussian kernel similarity, and ${D}_{F}$ is the drug functional similarity.

Similarly, the microbe integrated similarities matrix $M\in {R}^{{n}_{m}\times {n}_{m}}$ was calculated as follows:

7 $$M=\left\{\begin{array}{c}\left({M}_{GIP}+{M}_{F}\right)/2, if {M}_{F}\ne 0\\ {M}_{GIP}, if {M}_{F}=0\end{array}\right.$$

where ${M}_{GIP}$ is the drug Gaussian kernel similarity, and ${M}_{F}$ is the drug functional similarity.

### Constructing the heterogeneous network ${\varvec{N}}$

The microbe–drug adjacency matrix, drug integrated similarities matrix and microbe integrated similarities matrix can be joined together to form a whole matrix $N\in {R}^{\left({n}_{d}+{n}_{m}\right)\times \left({n}_{d}+{n}_{m}\right)}$:

8 $$N=\left[\begin{array}{cc}D& A\\ {A}^{T}& M\end{array}\right]$$

where ${A}^{T}$ represents $A{\prime}$ s transposition.

### Predicting microbe–drug associations by NNM

Currently, the convex optimization model includes nuclear norms, which are applied in many fields^28^. It has a globally optimal solution^11^. Therefore, the nuclear norm minimization of the heterogeneous network N can be expressed as:

9 $$\underset{N}{{\text{min}}}{\Vert N\Vert }_{*} \quad s.t. {N}_{ij}={A}_{ij}, \left(i,j\right)\in \Omega $$

where ${\Vert N\Vert }_{*}$ represents the nuclear norm of $N$, $\Omega$ is a set of known positions of elements.

We need to add restrictions to the model to make sure that the unknown elements fall within the range [0,1] since predicted scores for microbe–drug associations should be between [0,1]. This forecasting method is:

10 $$\underset{N}{{\text{min}}}{\Vert N\Vert }_{*} s.t. {\Vert {p}_{\Omega }\left(N\right)-{p}_{\Omega }\left(A\right)\Vert }_{F}\le \varepsilon $$

11 $${\left({p}_{\Omega }\left(N\right)\right)}_{ij}=\left\{\begin{array}{c}{N}_{ij}, if\left(i,j\right)\in \Omega \\ 0, otherwise\end{array}\right.$$

They are $\varepsilon$, which stands for measurement noise, ${\Vert \cdot \Vert }_{F}$, which stands for the Frobenius norm, and ${p}_{\Omega }$, which stands for the orthogonal mapping acting on $\Omega$. Then substituting regularized models for inequality constrained models:

12 $$\underset{N}{{\text{min}}}{\Vert N\Vert }_{*}+\frac{\alpha }{2}{\Vert {P}_{\Omega }\left(N\right)-{p}_{\Omega }\left(A\right)\Vert }_{F}^{2} \quad s.t. 0<{N}_{ij}<1$$

where $\mathrm{\alpha }$ is a variable that is learnable. The model can be optimized in the manner shown below by introducing the auxiliary matrix X, which was inspired by literature^29^:

13 $$\underset{N}{{\text{min}}}{\Vert N\Vert }_{*}+\frac{\alpha }{2}{\Vert {P}_{\Omega }\left(X\right)-{p}_{\Omega }\left(A\right)\Vert }_{F}^{2} \quad s.t. N=X, 0<{X}_{ij}<1$$

Then, minimize the enhanced Lagrange function to solve the problem:

14 $$\zeta \left(X,N,Y,\alpha ,\beta \right)={\Vert N\Vert }_{*}+\frac{\alpha }{2}{\Vert {P}_{\Omega }\left(X\right)-{p}_{\Omega }\left(A\right)\Vert }_{F}^{2}+{T}_{\gamma }\left({Y}^{T}\left(N-X\right)\right)+\frac{\beta }{2}{\Vert N-X\Vert }_{F}^{2}$$

where $Y$ is the Lagrange multiplier and $\beta >0$ is the penalty factor.

Following that, implement iterative solution. The matrix ${X}_{k+1}$ must first be calculated:

$${X}_{k+1}=arg\underset{0\le X\le 1}{{\text{min}}}\zeta \left(X,{N}_{k},{Y}_{k},\alpha ,\beta \right)$$

15 $$=arg\underset{0\le X\le 1}{{\text{min}}}\frac{\alpha }{2}{\Vert {P}_{\Omega }\left(X\right)-{p}_{\Omega }\left(A\right)\Vert }_{F}^{2}+{T}_{\gamma }\left({Y}^{T}\left(N-X\right)\right)+\frac{\beta }{2}{\Vert N-X\Vert }_{F}^{2}$$

The best answer to the Eq. (15) for $arg\underset{0\le X\le 1}{{\text{min}}}\zeta \left(X,{N}_{k},{Y}_{k},\alpha ,\beta \right)$ is ${X}^{*}$. Think of restrictions for the interval [0,1] that:

16 $${\left({X}_{k+1}\right)}_{ij}=\left\{\begin{array}{c}1, {X}_{ij}^{*}>1\\ {X}_{ij}^{*}, 0\le {X}_{ij}^{*}\le 1\\ 0, {X}_{ij}^{*}<0\end{array}\right\}$$

Update the matrix ${N}_{k+1}$ and correct other variables:

$${N}_{k+1}=arg\underset{N}{{\text{min}}}\zeta \left({X}_{k+1},N,{Y}_{k},\alpha ,\beta \right)$$

17 $$=arg\underset{N}{{\text{min}}}{\Vert N\Vert }_{*}+\frac{\beta }{2}{\Vert N-\left({X}_{k+1}-\frac{{Y}_{k}}{\beta }\right)\Vert }^{2}={\vartheta }_{\frac{1}{\beta }}\left({X}_{k+1}-\frac{{Y}_{k}}{\beta }\right)$$

18 $${\vartheta }_{\tau }(x)={\sum }_{i=1}^{{\theta }_{i}\ge \tau }\left({\theta }_{i}-\tau \right){\mu }_{i}{\nu }_{i}^{T}$$

where ${\vartheta }_{\tau }(x)$ is singular value contraction operator, ${\theta }_{i}$ is the singular values of $X$ which is larger than $\tau$, while ${\mu }_{i}$ and ${\nu }_{i}$ are the left and right singular vectors corresponding to ${\theta }_{i}$.

We can update the Lagrange multiplier ${Y}_{k+1}$ as follows by adjusting other variables:

19 $${Y}_{k+1}={Y}_{k}+\beta \left({N}_{k+1}-{X}_{k+1}\right)$$

Finally, the following information can be found in the prediction matrix ${A}_{1}$ for microbe–drug associations:

20 $${X}_{k}\to \left[\begin{array}{cc}{D}_{1}& {A}_{1}\\ {A}_{1}^{T}& {M}_{1}\end{array}\right]\to {A}_{1}$$

### Predicting latent microbe–drug associations by GAT

With the introduction of an attention-based design, the graph spatial network GAT performs node categorization for graph-structured data^24^. To determine the matrix $N$'s structure, we created a GAT model. First determines the attention score between any two nodes in the matrix $N$:

21 $${\alpha }_{ij}=\frac{exp\left({e}_{ij}\right)}{{\sum }_{k\in {N}_{i}}exp\left({e}_{ik}\right)}$$

22 $${e}_{ij}=LeakyRelu\left(a\left[W{h}_{i}||W{h}_{j}\right]\right)$$

23 $$LeakyRelu\left(x\right)=\left\{\begin{array}{l}x, \quad x>0\\ \mu x, \quad otherwise\end{array}\right.$$

where ${N}_{i}$ stands for the total number of nodes,$a$ is an attention coefficient, $W$ is a learnable linear transformation, and ${h}_{i}$ represents the feature vector of the node $i$, $\mu$ is the hypermeter and $||$ denotes the concatenation.

Consequently, each node's ultimate output feature is:

24 $${h}_{i}=relu\left(\sum_{j\in {N}_{i}}{\alpha }_{ij}W{h}_{j}\right)$$

The activation function, $relu$, is defined as follows:

25 $$relu\left(x\right)=\left\{\begin{array}{l}x, \quad x>0\\ 0, \quad otherwise\end{array}\right.$$

A low dimensional structural matrix $$X=\left[\begin{array}{c}{X}_{d}\\ {X}_{m}\end{array}\right]\in {R}^{\left({n}_{d}+{n}_{m}\right)\times l}$$ is produced by substituting $N$ into the previously mentioned GAT model, where ${X}_{d}$ and ${X}_{m}$, respectively, stand in for the drug nodes and microbial nodes in $N$. After a number of testing, we ultimately decided on MSE loss as the loss function for optimizing our model.

An improved random walk with restart (RWR) is implemented on D in response to literature^20^, allowing us to obtain a new matrix. Below is how the RWR was described:

26 $${s}_{i}^{l+1}=\lambda X{s}_{i}^{l}+\left(1-\lambda \right){\varepsilon }_{i}$$

27 $${\varepsilon }_{ij}=\left\{\begin{array}{l}1, \,\,if\, i=j\\ 0, \,\,otherwise\end{array}\right.$$

where ${\varepsilon }_{i}$ is the initial probability vector, $X$ is the matrix of transition probabilities, and $\uplambda$ is the restart probability. Similar to that, we might produce a novel matrix ${M}_{Z}$ by using the enhanced RWR on $M$.

As a result, by combining the drug matrix ${X}_{d}$, ${D}_{F}$, ${D}_{Z}$ and adjacency matrix $A$, influenced by literature^22^, we could create a new drug feature matrix ${Z}_{d}$ that looked like this:

28 $${Z}_{d}=\left[{X}_{d},{D}_{F},A,{D}_{Z},A\right]$$

Similarly, we could create the following new microbe feature matrix:

29 $${Z}_{m}=\left[{X}_{m},{A}^{T},{M}_{F},{{A}^{T},M}_{Z}\right]$$

Finally, we employ dot product to derive a microbe–drug association predictive score ${A}_{2}$:

30 $${A}_{2}=swich\left({Z}_{d}\left({d}_{i}\right)\cdot{{Z}_{m}\left({m}_{j}\right)}^{T}\right)$$

31 $$swich=xSigmoid\left(\beta x\right)$$

where $swich$ is an activation function, $\beta$, a learnable parameter, which is typically set it to 1, ${Z}_{d}\left({d}_{i}\right)$ indicates the ${i}_{th}$ row of ${Z}_{d}$ and ${Z}_{m}\left({m}_{j}\right)$ represents the ${j}_{th}$ row of ${Z}_{m}$.

### Final predicted score of microbe–drug associations

The weighted arithmetic mean approach can be used to combine the prediction matrix ${A}_{1}$ acquired through NNM and the prediction matrix ${A}_{2}$ generated through GAT, resulting in the following final forecast matrix ${A}^{*}$ of microbe–drug associations:

32 $${A}^{*}=\lambda {A}_{1}+\left(1-\lambda \right){A}_{2}$$

where $\lambda$ is the weight value.

## Experiments and results

In this section, we first carried out sensitive parameter analysis to get the optimum performance out of the model. Then, six state-of-the-art methods would be picked to contrast with NMGMDA. Finally, in order to confirm the validity of our model, we have chosen two typical microbes and drugs, respectively.

### Parameter sensitivity analysis

Three pieces make up the NMGMDA model. $\alpha$ and $\beta$ in formula (14) are two crucial parameters in NNM. Dimension $l$ and learning rate ${l}_{r}$ are the two most important factors in GAT.The weight value $\lambda$ is an important parameter in the final prediction formula (32). In this section, to find the appropriate settings and ensure the independence of the training sets and test sets, we initially Randomly picked 20% of the associations are known and 20% are unknown for the training sets, with the remaining sets being test sets. Next, we utilized fivefold CV experiments with the MDAD database and ensure each of the experiments is independent.

In NNM, we decided to conduct joint tests and altered $\alpha$ and $\beta$ from $\left\{\mathrm{0.1,1},\mathrm{10,100,1000}\right\}$ and conduct joint experiments.Then, using a fivefold CV experiment, we determined the area under curve (AUC) and the area under the precision-recall curve (AUPR) of these parameter combinations. The findings are displayed in Table 2. Table 2 shows that the AUC and AUPR outcomes obtained by NMGMDA are both at their best when $\alpha$ and $\beta$ have values of 100 and 1, respectively.

**Table 2:**
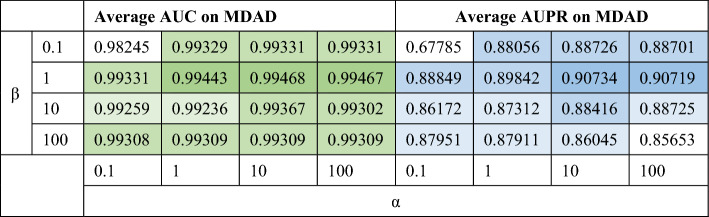
The AUC and AUPR values on different $\alpha$ and $\beta$ on MDAD database.

In GAT, we decided to adjust the dimension $l$ changed from $\left\{\mathrm{16,32,64,128}\right\}$ and the learning rate ${l}_{r}$ changed from $\left\{\mathrm{0.1,0.05,0.01,0.005,0.001}\right\}$.

**Figure 2:**
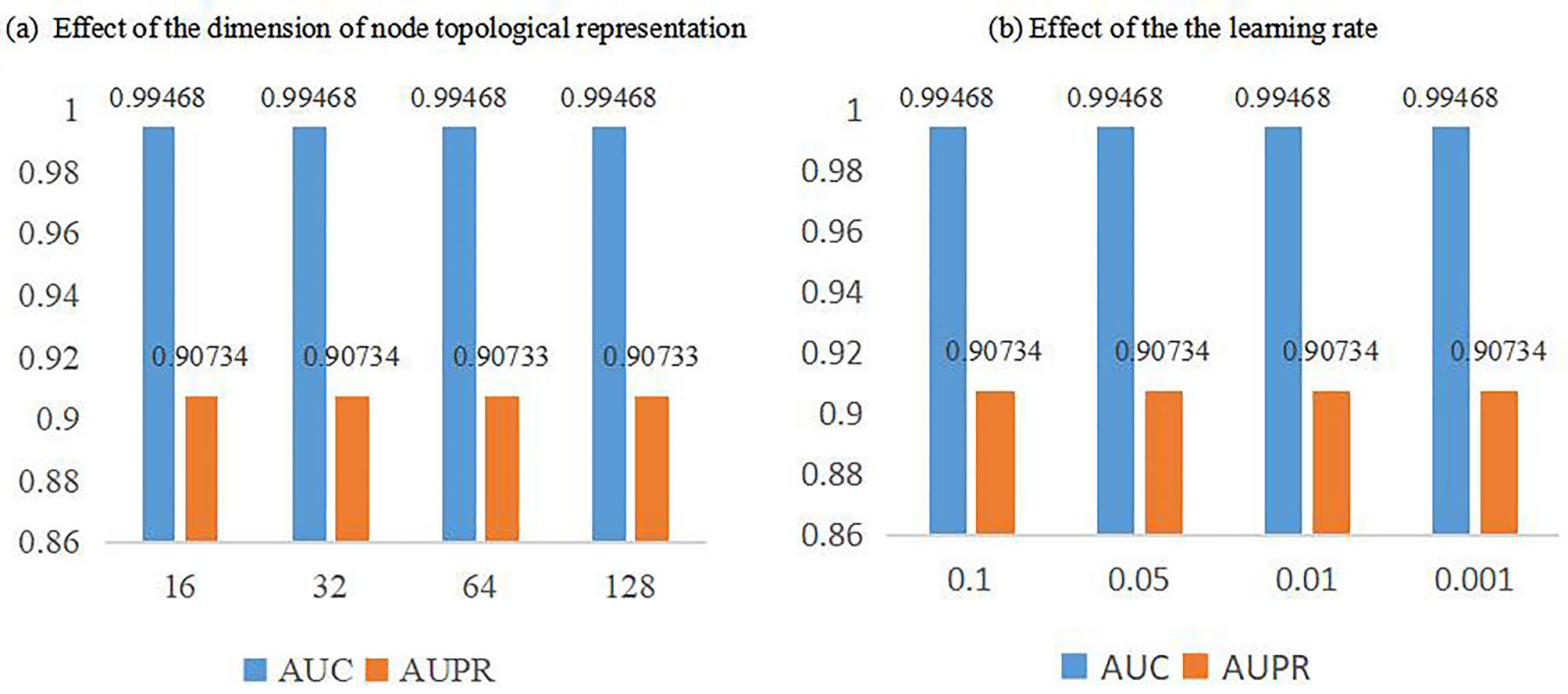
The AUC and AUPR values on different dimension of node topological representation and learning rate on MDAD database.

Figure 2 makes it clear that no substantial changes to the outcome were caused by changing any particular factors. We choose 32 as the dimension of node topological representation $l$ since it has a little better AUPR value than 64 or 128 dimensions. In line with typical learning models, the learning rate ${l}_{r}$ was set at 0.01.

**Figure 3:**
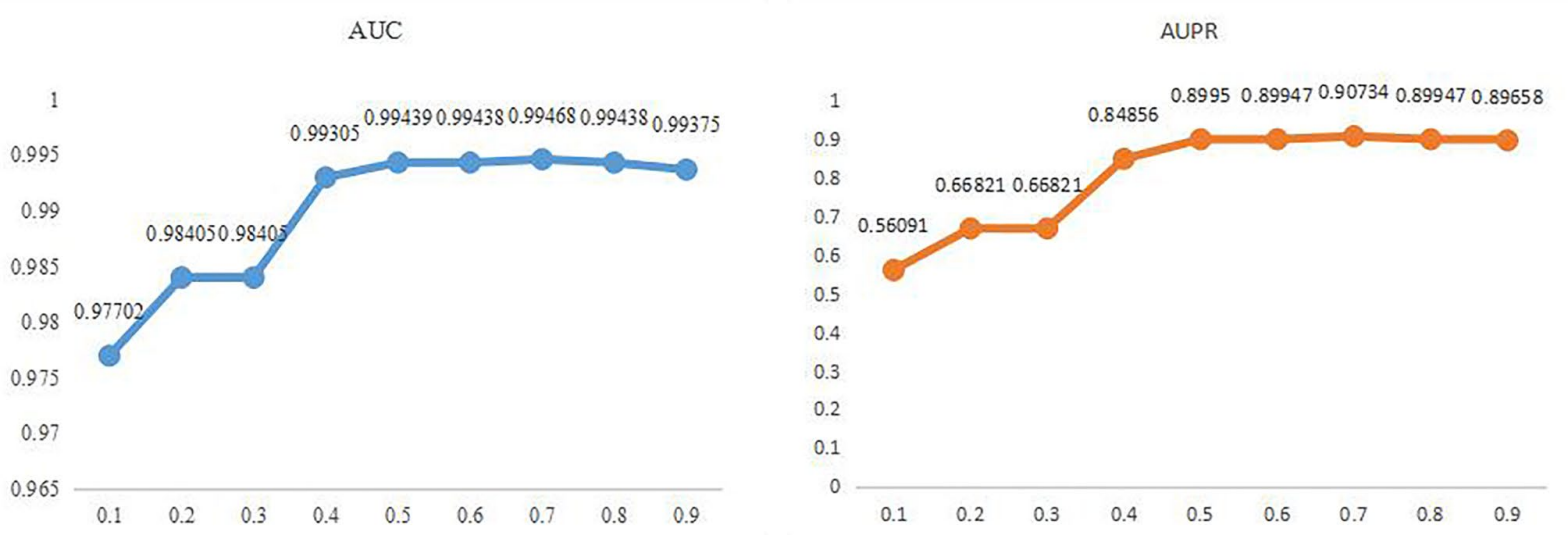
The AUC and AUPR values on different weight value on MDAD database.

Finally, the results are displayed in Fig. 3 for parameter $\lambda$ in formula (32), where we estimate the impact of the $\lambda$ altered from $\left\{\mathrm{0.1,0.2,0.3,0.4,0.5,0.6,0.7,0.8,0.9}\right\}$ for the fivefold on MDAD. Which makes it clear that NMGMDA, with $\lambda$ set to 0.7, may get the maximum AUC and AUPR values.

After comparing the performance on different hyperparameters by testing, the final parameters we selected are $\alpha$ = 100, $\beta$ = 1, $l$ = 32, ${l}_{r}$ = 0.01 and $\lambda$ = 0.7.

### Comparison with advanced methods

In this case, taking into account the dearth of microbial drug association prediction methods, we would first contrast NMGMDA with a few standard approaches for link prediction issues, such as HMDAKATZ^14^, HMDA-Pred^30^, LAGCN^31^, MNNMAD^32^ and GSAMDA^20^, etc.

Here, considering the limited availability of microbial drug association prediction methods, we would first compare NMGMDA with some representative methods for link prediction problems such as HMDAKATZ^14^, HMDA-Pred^30^, LAGCN^31^, MNNMAD^32^ and GSAMDA^20^, etc. One of them, HMDAKATZ, predicted the association between microbes and drugs using the KATZ algorithm as a foundation. For the prediction of microbe–disease associations, HMDA-Pred is a novel computer model based on multi-data integration and network consistency projection. LAGCN is a complete end-to-end graph based deep learning method, which forecast the associations between drugs and diseases. By using a Matrix Nuclear Norm approach on data on known microbes and diseases, MNNMAD is a method for predicting microbe–disease relationships. Based on a graph attention network and sparse auto-encoder, GSAMDA offered a unique computer model for forecasting probable microbe–drug interactions.

**Figure 4:**
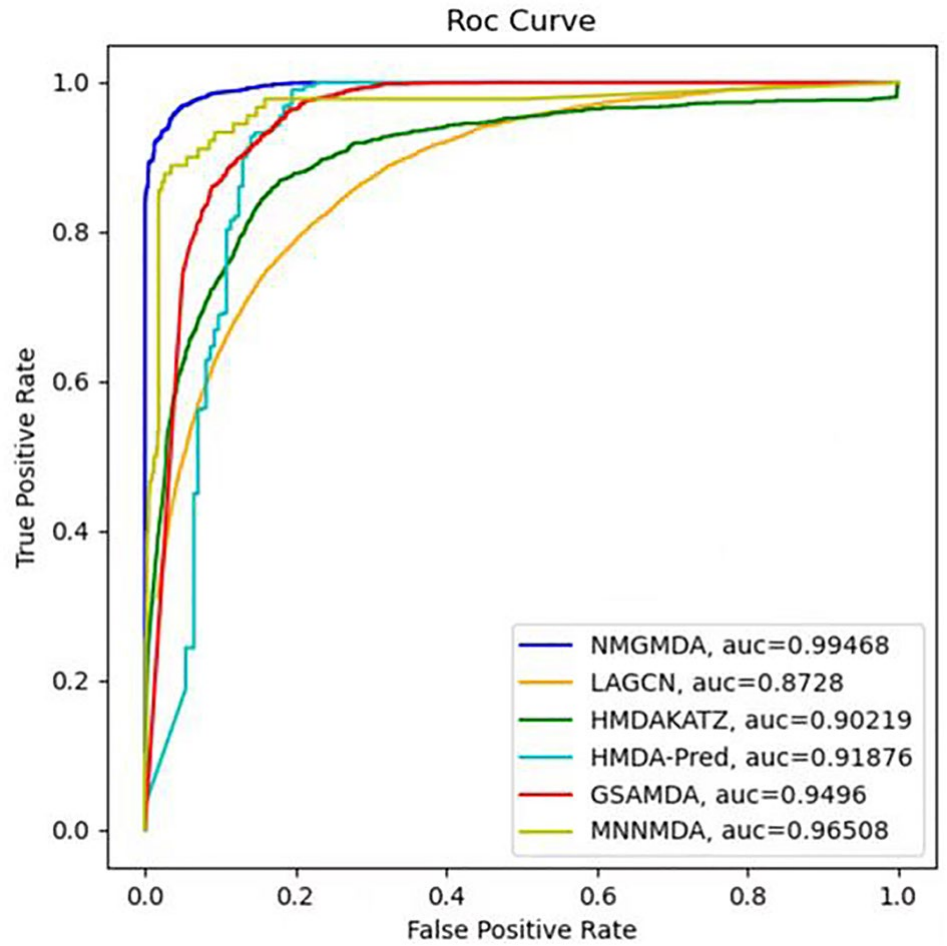
ROC curves based on the MDAD database for six competitive methods.

We tested these techniques using their default settings and compared them using the fivefold CV experiment. AUC and AUPR values are used as indicators to evaluate the performance of NMGMDA, and the database we utilize is MDAD and aBioflm. The outcome was displayed in Table 3 and Fig. 4. Our suggested NMGMDA model has the greatest prediction performance of all the methods.

**Table 3:** The AUCs and AUPRs of compared methods based on databases MDAD and aBioflm under fivefold CV.

Methods	AUC		AUPR	
	MADA	aBioflm	MDAD	aBioflm
HMDAKATZ	0.90118 ± 0.00101	0.90023 ± 0.00212	0.23271 ± 0.00685	0.30669 ± 0.00771
LAGCN	0.86883 ± 0.00703	0.86413 ± 0.01091	0.35712 ± 0.00514	0.36715 ± 0.00556
HMDA-Pred	0.91776 ± 0.00297	0.91756 ± 0.00402	0.02361 ± 0.00093	0.02848 ± 0.00069
MNNMDA	0.96306 ± 0.00202	0.93156 ± 0.00223	0.18920 ± 0.00561	0.19621 ± 0.00782
GSAMDA	0.94917 ± 0.00053	0.93073 ± 0.01206	0.44363 ± 0.00072	0.45103 ± 0.00513
NMGMDA	0.99468 ± 0.00016	0.99467 ± 0.00017	0.90734 ± 0.00019	0.90330 ± 0.00018

### Case study

To test the NMGMDA model's real prediction power, we chose two well-known drugs—Ciprofloxacin and Moxifloxaxin—as well as two common microbes—Human immunodeficiency virus type 1 and Mycobacterium tuberculosis—for case studies.

Ciprofloxacin is an organic molecule with excellent bactericidal effect and broad-spectrum antibacterial activity^33^. It has shown to be a successful treatment for both acute and chronic urinary tract infections, as well as a variety of systemic infections^34^. Staphylococcus aureus^35^, Haemophilus influenzae^36^ and Stenotrophomonas maltophilia^37^ are all susceptible to its antibacterial properties. Based on the predicted score, ranked the Ciprofloxacin-related microbes scores from highest to lowest, and chose the top 20 microbes for validation after deleting the 10 associations that are currently on MDAD database. As indicated in Table 4, 19 of the top 20 anticipated microbes connected to Ciprofloxacin have been verified by published research in PubMed. Moreover, Moxifloxacin^39^ belongs to the quinolone drugs class, which mostly used to treat infections of the skin and soft tissues in adults as well as upper and lower respiratory tract infections^38,39^. According to the literature^40^, Moxifloxacin is an effective treatment for Stenotrophomonas maltophilia keratitis. As indicated in Tables 5, after removing the 4 known associations on MDAD database, we discovered 17 microbes that had been verified by PubMed literature among the top 20 predicted microbes associated with moxifloxacin.

**Table 4:** The top 20 Ciprofloxacin associated candidate microbes on MDAD.

Microbe	Evidence	Microbe	Evidence
Enteric bacteria and other eubacteria	PMID: 36682905	*Acinetobacter baumannii*	PMID: 34098109
*Micrococcus luteus*	PMID: 3010848	*Klebsiella pneumoniae*	PMID: 29055688
*Bacillus anthracis*	PMID: 23569822	*Burkholderia pseudomallei*	PMID: 35972245
*Helicobacter pylori*	PMID: 24837413	*Candida albicans*	PMID: 35404123
*Listeria monocytogenes*	PMID: 34068252	*Vibrio anguillarum*	PMID: 36735199
*Burkholderia cenocepacia*	PMID: 25267676	*Actinomyces oris*	Unconfrmed
*Burkholderia multivorans*	PMID: 34524889	*Bacillus cereus*	PMID: 26358183
*Mycobacterium avium*	PMID: 30012773	*Vibrio vulnificus*	PMID: 28971862
*Vibrio harveyi*	PMID: 27247095	*Streptococcus mutans*	PMID: 33402618
*Klebsiella planticola*	PMID: 36452290	*Streptococcus epidermidis*	PMID: 34948159

The top 10 microbes are listed in the first column, while the top 11–20 microbes are listed in the third column.

**Table 5:** The top 20 Moxifoxacin associated candidate microbes on MDAD.

Microbe	Evidence	Microbe	Evidence
*Staphylococcus aureus*	PMID: 33936821	*Burkholderia multivorans*	Unconfrmed
*Escherichia coli*	PMID: 34653694	*Providencia stuartii*	Unconfrmed
*Pseudomonas aeruginosa*	PMID: 23662986	*Burkholderia cenocepacia*	PMID: 33120688
*Bacillus subtilis*	PMID: 30036828	*Mycobacterium tuberculosis*	PMID: 33951360
*Staphylococcus epidermidis*	PMID: 15718814	*Enterococcus faecalis*	PMID: 26349832
*Streptococcus mutans*	PMID: 29160117	*Vibrio harveyi*	Unconfrmed
*Staphylococcus epidermis*	PMID: 31516359	*Klebsiella planticola*	PMID: 32577260
*Salmonella enterica*	PMID: 34439014	*Listeria monocytogenes*	PMID: 18299415
*Micrococcus luteus*	PMID: 16152924	*Candida glabrata*	PMID: 20455400
*Shigella flexneri*	PMID: 28483960	*Streptococcus pneumoniae*	PMID: 22407042

The top 10 microbes are listed in the first column, while the top 11–20 microbes are listed in the third column.

Regarding microbes, the first microbe is Human immunodeficiency virus type 1 (HIV-1), which is a virus capable of attacking the immune system in humans, and causes AIDS, an extremely dangerous infectious illness^41^. HIV-1 has been widely studied in relation to various medicines. Saquinavir, for instance, has been shown to be an effective treatment for HIV-1-infected individuals who have diarrhea and/or wasting syndrome by Hervé Trout^42^. According to literature^43^, the first-line protease inhibitor that is generally suggested in the initial treatment regimen for people with HIV-1 infection is lopinavir/ritonavir. After removing the 26 known associations on MDAD database, we discovered 16 (see Table 6) drugs that had been validated by PubMed literatures among the top 20 anticipated microbes associated with Human immunodeficiency virus type 1. Mycobacterium tuberculosis is the second microbes used in the case study. Mycobacterium tuberculosis is the pathogen that causes tuberculosis^44^, and many microbes, including ciprofloxacin^45^ and triclosan^46^, have been shown to be associated with it. After removing the 14 known associations on MDAD database, Table 7 indicates that of the top 20 candidate drugs, 14 were linked to Mycobacterium tuberculosis.

**Table 6:** The top 20 Human immunodeficiency virus type 1 associated candidate drugs on MDAD.

Drug	Evidence	Drug	Evidence
Delavirdine	PMID: 8807058	Ceftazidime	PMID: 7979856
Nelfinavir	PMID: 10223697	Amikacin	PMID: 16371246
LL-37	PMID: 24821067	Tobramycin	PMID: 27387258
Farnesol	PMID: 22677126	Cefixime	PMID: 9677171
Vancomycin	PMID: 32328074	Curcumin	PMID: 19345695
Epigallocatechin Gallate	PMID: 11684313	N-Acetylcysteine	PMID: 32796068
Toremifene	Unconfrmed	IDR-1018	Unconfrmed
Norspermidine	Unconfrmed	Metronidazole	PMID: 18444793
Hamamelitannin	PMID: 8693037	Terpinene-4-ol	Unconfrmed
Ciprofloxacin	PMID: 9566552	Carvacrol	PMID: 32461309

The top 10 drugs are listed in the first column, while the top 11–20 drugs are listed in the third column.

**Table 7:** The top 20 *Mycobacterium tuberculosis* associated candidate drugs on MDAD.

Drug	Evidence	Drug	Evidence
Curcumin	PMID:27012592	Norspermidine	Unconfrmed
Epigallocatechin gallate	PMID:33463343	Moxifloxacin	PMID: 33951360
Metronidazole	PMID:18491971	Pleurocidin	Unconfrmed
BMAP-28	Unconfrmed	Esculetin	Unconfrmed
Vancomycin	PMID:33508482	Gentamicin	PMID: 36258995
Ceftazidime	PMID:11527042	Amikacin	PMID: 34314673
Cefixime	Unconfrmed	Silver nanoparticles	PMID: 32280217
Farnesol	PMID:16041726	Carboxymethyl chitosan	PMID: 30007629
Azithromycin	PMID: 32781595	Toremifene	Unconfrmed
LL-37	PMID: 26351280	Indole	PMID: 34383995

The top 10 drugs are listed in the first column, while the top 11–20 drugs are listed in the third column.

In conclusion, these two sets of case studies further demonstrate how the NMGMDA model may anticipate the association between microbes and drugs.

## Discussion and conclusion

The association between drugs and microbes has enormous significance for the treatment of diseases, according to biomedical studies. Consequently, a powerful computational prediction model could help researchers find additional microbe–drug associations and improve illness therapy.

By combining the NNM and GAT frameworks, we suggested a unique model in this study called NMGNAD to forecast potential microbe–drug associations. In NMGNAD, we first combined the drug similarity network, the known microbe–drug associations, and the similarity and association information between nodes to create a new microbe–drug heterogeneous network. The correlation scores between microbes and drugs were then predicted using the NNM model and the GAT model. In order to get the forecast results, we weighted average these two anticipated scores. According to experimental findings, NMGMAD outperformed state-of-the-art methods and produced acceptable case study outcomes.

Although NMGMDA can produce good prediction performance, there are still certain restrictions. First off, a few drug names in both databases are not accessible now, and the fact that they are no longer being updated will reduce the number of known connections that are available and have an impact on how the model is used in practice. Thus, we might think about creating a microbe–drug database that is more extensive. Then, to increase the precision of model predictions, we can think about adding more biological data to enhance the characteristics of drugs and microbes, such as data on drug side effects, data on the relationship between germs and diseases, and data on the association between drugs and diseases.

## Data Availability

The original contributions presented in the study are included in the article, further inquiries can be directed to the corresponding authors.
